# Fermi surface and pseudogap in highly doped Sr_2_IrO_4_

**DOI:** 10.1038/s41535-025-00817-9

**Published:** 2025-10-03

**Authors:** Y. Alexanian, A. de la Torre, S. McKeown Walker, M. Straub, G. Gatti, A. Hunter, S. Mandloi, E. Cappelli, S. Riccò, F. Y. Bruno, M. Radovic, N. C. Plumb, M. Shi, J. Osiecki, C. Polley, T. K. Kim, P. Dudin, M. Hoesch, R. S. Perry, A. Tamai, F. Baumberger

**Affiliations:** 1 Department of Quantum Matter Physics, University of Geneva, Geneva, Switzerland; 2 Department of Physics, Northeastern University, Boston, MA USA; 3 Quantum Materials and Sensing Institute, Northeastern University, Burlington, MA USA; 4 Laboratory of Advanced Technology, University of Geneva, Geneva, Switzerland; 5 GFMC, Departamento de Física de Materiales, Universidad Complutense de Madrid, Madrid, Spain; 6 Swiss Light Source, Paul Scherrer Institut, Villigen, Switzerland; 7 Center for Correlated Matter and School of Physics, Zhejiang University, Hangzhou, China; 8 MAX IV Laboratory, Lund University, Lund, Sweden; 9 Diamond Light Source, Harwell Campus, Didcot, UK; 10 Synchrotron SOLEIL, Gif sur Yvette, France; 11 Deutsches Elektronen-Sychrotron DESY, Hamburg, Germany; 12 ISIS Pulsed Neutron and Muon Source, STFC Rutherford Appleton Laboratory, Harwell Campus, Didcot, UK; 13 London Centre for Nanotechnology and Department of Physics and Astronomy, University College London, London, UK

**Keywords:** Electronic properties and materials, Superconducting properties and materials

## Abstract

The fate of the Fermi surface in bulk electron-doped Sr_2_IrO_4_ remains elusive, as does the origin and extension of its pseudogap phase. Here, we use high-resolution angle-resolved photoelectron spectroscopy (ARPES) to investigate the electronic structure of Sr_2−*x*_La_*x*_IrO_4_ up to *x* = 0.2, a factor of two higher than in previous work. We find that the antinodal pseudogap persists up to the highest doping level, and thus beyond the sharp increase in Hall carrier density to ≃ 1 + *x* recently observed above x* ≃ 0.16^1^. This suggests that doped iridates host a unique phase of matter in which a large Hall density coexists with an anisotropic pseudogap, breaking up the Fermi surface into disconnected arcs. The temperature boundary of the pseudogap is T* ≃ 200 K for *x* = 0.2, comparable to cuprates and to the energy scale of short range antiferromagnetic correlations in cuprates and iridates.

## Introduction

The pseudogap (PG) in hole-doped cuprates is one of the most enigmatic properties of correlated electron systems. A pragmatic definition of a PG, adopted throughout this article, is the existence of a sharp suppression of spectral weight at low energy scales. ARPES experiments established that the cuprate PG is anisotropic and selectively suppresses the low-energy spectral weight near (*π*, 0), leaving apparent Fermi arcs extending out from the node along the Brillouin zone diagonal^2,3^. However, the origin of the PG and its relation with the rich phase diagram of cuprates remain controversial, not least because there is little thermodynamic evidence for a genuine phase transition at the critical doping p* and temperature T* where the PG closes^4–7^. When superconductivity is suppressed in high magnetic fields, several cuprate families show a strong peak in the electronic specific heat near p*, in some cases accompanied by a $\log (1/T)$ dependence at low temperatures. Around the same doping level, the Hall carrier density increases from the doping *p* to 1 + *p*^8,9^. Whether these signatures are caused by a quantum critical point associated with the closure of the PG remains debated.

The recent observation by Hsu et al. of similar anomalies in the electronic specific heat and Hall density in the electron-doped iridate Sr_2−*x*_La_*x*_IrO_4_ provides complementary insight into these open questions^1^. Undoped Sr_2_IrO_4_ is a single-band antiferromagnetic (AF) insulator, commonly described as a pseudospin *J*_eff_ = 1/2 Mott state^10–12^, although other interpretations have been put forward^13^. A minimal model of electron-doped Sr_2_IrO_4_ remarkably resembles that of hole-doped cuprates^14^. ARPES experiments at low La (i.e., electron) doping revealed a PG with the same anisotropy in momentum space known from hole-doped cuprates^15–17^. Crucially, though, Sr_2−*x*_La_*x*_IrO_4_ shows no signs of superconductivity up to the highest doping of *x* = 0.2 investigated so far^18^. On the other hand, short-range AF spin correlations in Sr_2−*x*_La_*x*_IrO_4_ closely reflect spin excitations in cuprates^19–22^, although no charge or spin orders were found in iridates. These observations prompted suggestions that the PG in iridates is driven by magnetic fluctuations^1,15^.

Hsu et al. interpreted the anomalies in specific heat and Hall density as a signature of the closure of the PG at a critical La doping x* ≃ 0.16^1^. This is qualitatively consistent with an ARPES study of K surface-doped Sr_2_IrO_4_ that found a transition from a pseudogapped regime to a conventional large Fermi surface (FS) around a K coverage of ~ 0.85 monolayer (ML)^23^. However, it is unclear whether the K/AF-insulator interface is representative of highly bulk-doped samples, which are metallic and paramagnetic. In bulk electron-doped samples, there is thus far no direct evidence for a closure of the PG.

Here, we report high-resolution ARPES data from the same batch of Sr_2−*x*_La_*x*_IrO_4_ samples studied by Hsu et al.^1^. We find that the electronic structure evolves smoothly across x*. Quasiparticle coherence increases monotonously with doping while the nodal Fermi velocity is largely constant. Most importantly, the PG remains open up to at least *x* = 0.2 and thus beyond the Hall density crossover reported by Hsu et al.^1^ at x* ≃ 0.16. This demonstrates that a pseudogapped state can coexist with a Hall carrier density of ≃ 1 + *x*, conventionally interpreted as a large closed Fermi surface. We further show that for *x* = 0.2, the PG vanishes around T* ≃ 200 K, comparable to T* of cuprates and to the Néel transition temperature of the undoped compounds.

## Results

### Doping dependence

**Fig. 1:**
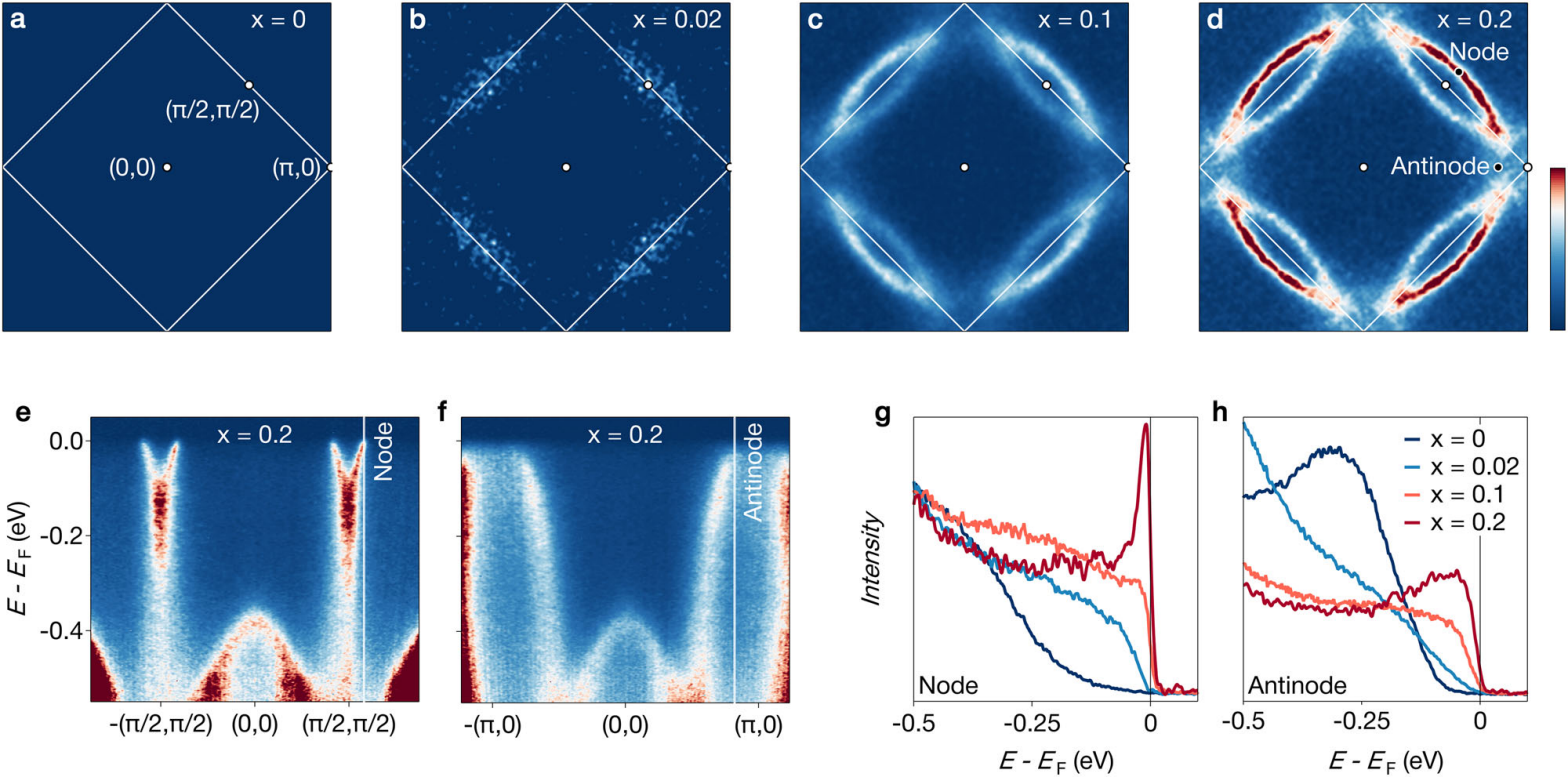
Collapse of the spin-orbit driven Mott insulating ground state and emergence of the pseudogap state in Sr_2−*x*_La_*x*_IrO_4_. **a-d** Fermi surfaces for *x* = 0, *x* = 0.02, *x* = 0.1, and *x* = 0.2, respectively. Data were measured at *T* ≃ 50 K (**a**, **b**) and *T* ≃ 10 K (**c**, **d**) with a photon energy *hν* = 100 eV and have been fourfold rotationally averaged. **e**, **f** ARPES band dispersion of Sr_1.8_La_0.2_IrO_4_ along the nodal (0, 0) − (*π*, *π*) and antinodal (0, 0) − (*π*, 0) directions, illustrating the nodal-antinodal dichotomy. **g**, **h** Doping dependence of energy distribution curves (EDCs) at the nodal and antinodal positions indicated in (**d–f**).

Figure 1 illustrates the doping dependence of the electronic structure of Sr_2−*x*_La_*x*_IrO_4_ from the pristine compound to *x* = 0.2. Incoherent spectral weight near the Fermi energy *E*_F_ first appears at *x* = 0.02. However, a defined PG state with coherent Fermi arcs stretching from the nodal (0, 0) − (*π*, *π*) direction and evolving into incoherent antinodal excitations only emerges at *x* = 0.1. The transition from an insulating to a pseudogapped state and the persistence of the nodal-antinodal dichotomy up to *x* = 0.2 are evident from the energy distribution curves (EDCs) in Fig. 1g, h. EDCs at the antinode show that although the leading edge of spectral weight shifts closer to *E*_F_ with increasing doping, a gap persists across the entire doping range. In contrast, nodal EDCs display the Fermi-Dirac cutoff of ungapped excitations for *x* = 0.1 and *x* = 0.2. Notably, the quasiparticle peak – nearly absent for *x* = 0.1 – becomes well-defined at *x* = 0.2. This is a signature of a striking increase in the nodal quasiparticle coherence with doping. These ARPES signatures of the PG state of Sr_2_IrO_4_ are highly reminiscent of hole-doped cuprates. Yet there are two key differences. First, the FS in Sr_2_IrO_4_ is electron-like and centered at (0,0), contrasting with the hole-like FS at (*π*, *π*) of the cuprates. Second, a doubling of the in-plane unit cell arising from a rotation of the oxygen octahedra causes a back folding of the FS in Sr_2−*x*_La_*x*_IrO_4_^15,24,25^.

The emergence of a distinct coherent nodal quasiparticle peak from the broad incoherent features dominating the spectra at half-filling is also a key feature of pseudogapped cuprates. There, it appears at doping levels deep into the superconducting state but below the critical doping where the PG terminates^26,27^. This indicates that our highly doped iridates are approaching the pseudogap doping boundary, and that further doping could lead to the predicted ungapped but still nodal-antinodal differentiated state^28^. It further highlights the conspicuous lack of superconductivity throughout the iridate pseudogap phase.

**Fig. 2:**
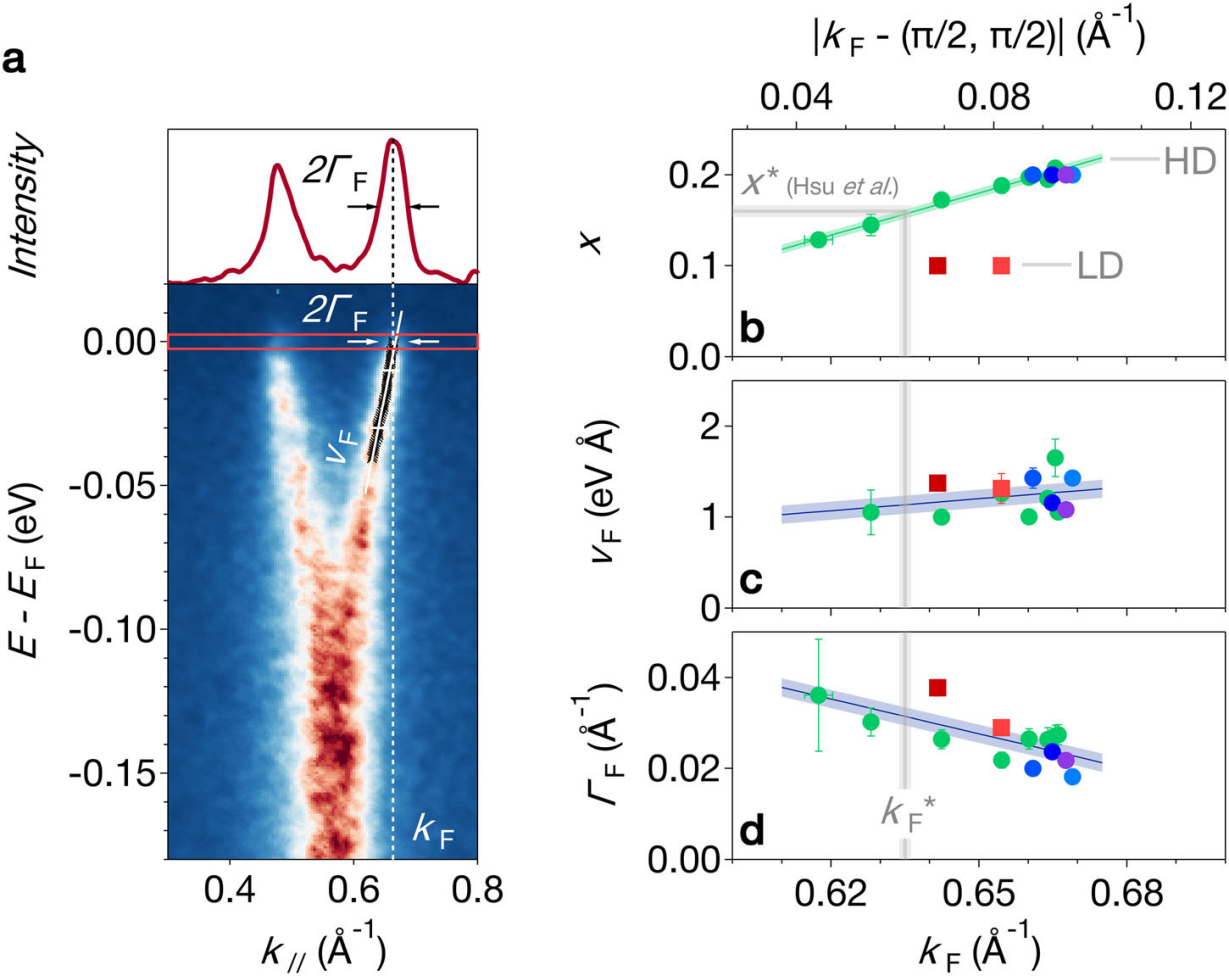
Continuous evolution of the electronic structure of Sr_2−*x*_La_*x*_IrO_4_ with doping. **a** Experimental definition of Fermi momentum *k*_F_, Fermi velocity *v*_F_, and scattering rate at the Fermi energy *Γ*_F_. See methods for details. **b** Lanthanum concentration *x* versus Fermi momentum *k*_F_. The green markers represent experimental data measured on a single HD sample which showed a smooth spatial variation of the La doping (see Supplementary Information A). A linear fit of these data (green line) defines the critical Fermi momentum ${k}_{{\rm{F}}}^{* }$ corresponding to the critical chemical doping x* of Ref. ^1^ (light grey line). *k*_F_ measurements on other samples are represented by different colored markers - red squares for lightly doped (LD) and blue/purple dots for highly doped (HD) samples. **c**, **d** Fermi velocity *v*_F_ and scattering rate at the Fermi energy *Γ*_F_ as a function of the Fermi momentum *k*_F_. Doping values *x* in (**b**) have been averaged over the ARPES beam spot diameter of 10 *μ*m and are given with their respective standard deviations, which are often smaller than the marker size. Error bars of *k*_F_, *v*_F_ and *Γ*_F_ in (**b**–**d**) represent standard deviations of the fits and are often smaller than the marker size.

**Fig. 3:**
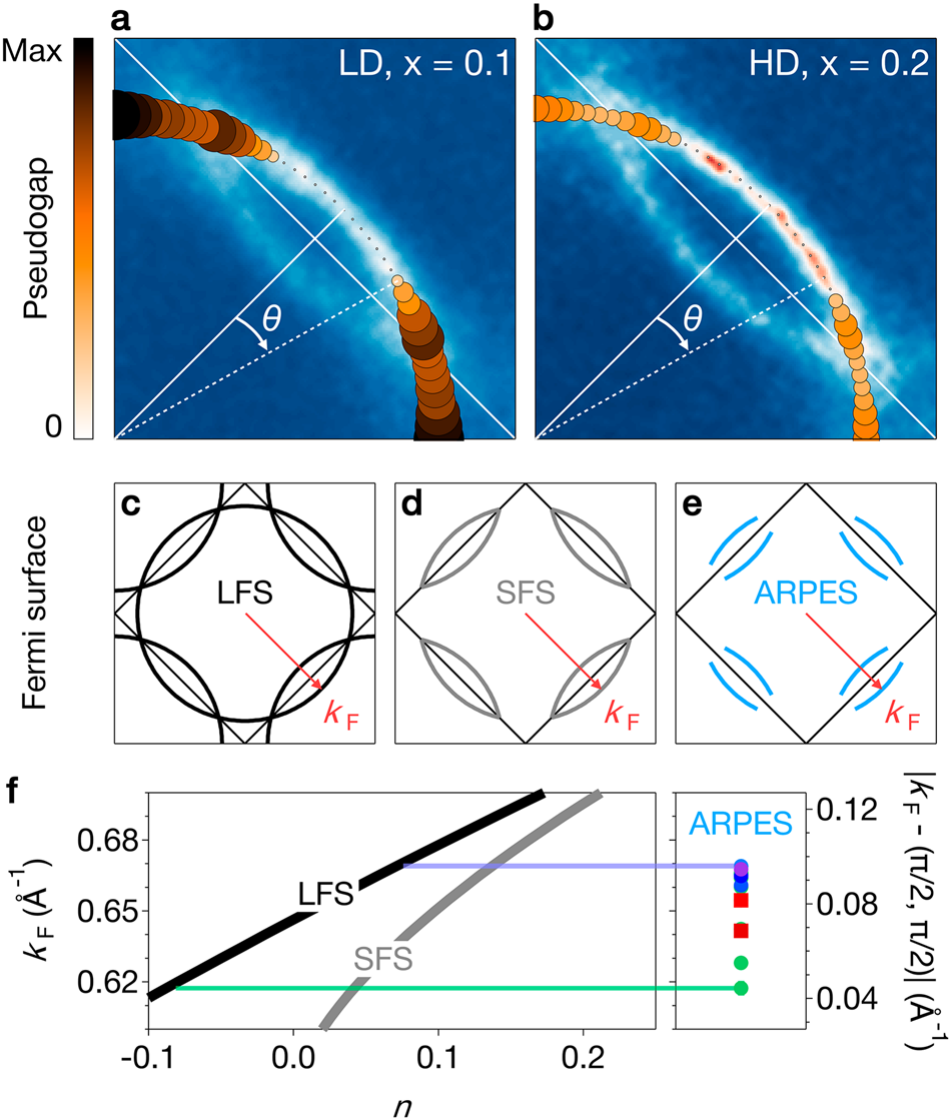
Pseudogap state of Sr_2−*x*_La_*x*_IrO_4_. **a**, **b** Spectral weight suppression in the PG (represented by both marker size and color) overlaid on a quadrant of the Fermi surface for *x* = 0.1 and *x* = 0.2. A common scale based on the larger value observed for *x* = 0.1 is used for both panels. **c** Schematic representation of the large (LFS) and **d** small (SFS) Fermi surface scenarios. **e** Sketch of the actual Fermi surface measured by ARPES. **f** Left: carrier density per Ir *n* versus Fermi momentum *k*_F_ in the large (black line) and small (grey line) scenarios. Right: range of *k*_F_ measured by ARPES in this study for samples with *x* between 0.1 and 0.2, as shown in Fig. 2b–d.

Figure 2 quantifies the evolution of the electronic structure of Sr_2−*x*_La_*x*_IrO_4_ with doping. To this end, we measured several samples grown with two different procedures (see Methods): lightly doped (nominal doping *x* = 0.1) and highly doped (nominal doping *x* = 0.2) samples, referred to as LD and HD, respectively. Crucially, our HD samples come from the same batch as those measured by Hsu et al.^1^, allowing for a straightforward comparison of results. We determined the precise La content *x* in both sets with energy-dispersive X-ray spectroscopy (EDX). These measurements revealed a slow variation in *x* near the edges of some HD samples. We exploit this smooth gradient to track the doping dependence of the nodal electronic structure by combining the EDX analysis with spatially resolved *μ*-spot ARPES measurements (see Supplementary Information A). We first determine the nodal Fermi wave vector *k*_F_(*x*) (shown as *x*(*k*_F_) in Fig. 2b) from fits to momentum distribution curves (MDCs). This reveals a clear dichotomy between LD and HD samples, implying that in one or both cases, the itinerant carrier density differs from the La concentration. We will discuss this point in more detail in Fig. 3. Importantly, though, for the HD samples, also studied by Hsu et al.^1^, we observe a clean linear evolution of *k*_F_ with *x*, without any discontinuity at x* ≃ 0.16. This allows for a precise determination of the critical nodal ${k}_{{\rm{F}}}^{* }$ corresponding to the critical doping x* identified in Ref. ^1^.

We next determine the nodal Fermi velocity *v*_F_ and the scattering rate at the Fermi energy *Γ*_F_ from MDC fits (see Methods for details of the analysis). When plotted against *k*_F_, rather than *x* (Fig. 2c, d), *v*_F_ and *Γ*_F_ of LD and HD samples collapse onto a single curve. This shows that *k*_F_ is a more reliable indicator of the electronic state than the La concentration.

As *k*_F_ and thus the effective electron doping increases, we only observe a small gradual increase in *v*_F_, with no abrupt changes detected at ${k}_{{\rm{F}}}^{* }$. The reduction of the scattering rate with increasing *k*_F_ is more pronounced, in line with the significant rise in nodal quasiparticle coherence observed between the LD, *x* = 0.1 and the HD, *x* = 0.2 samples (Fig. 1g). Fig. 2c, d suggest a continuous evolution of *v*_F_ and *Γ*_F_ across the critical doping ${k}_{{\rm{F}}}^{* }$. However, we presently have few data points only with ${k}_{{\rm{F}}} < {k}_{{\rm{F}}}^{* }$ for which the Fermi velocity and scattering rate remain well defined. We thus cannot fully exclude a more pronounced change at ${k}_{{\rm{F}}}^{* }$.

Hsu et al. reported a strong enhancement of the electronic specific heat coefficient *γ* of Sr_2−*x*_La_*x*_IrO_4_ over an extended doping range of *x* ≃ 0.12−0.17. They further point out that their data show no signs of a logarithmic divergence of the specific heat, typical for quantum critical systems^1^. In a conventional quasi-2D metal, *γ* ∝ m* ∝ 1/*v*_F_ where m* is the quasiparticle effective mass. An enhancement of *γ* thus corresponds to an enhanced effective mass m* and a correspondingly reduced Fermi velocity *v*_F_. Our data are difficult to reconcile with such an interpretation of the measured electronic specific heat. Specifically, Fig. 2c shows that the nodal Fermi velocity remains nearly constant from ${x}^{* }/{k}_{{\rm{F}}}^{* }$ up to the highest doping of *x* = 0.2. In contrast, Hsu et al. report a roughly 6-fold decrease of *γ* over the same doping range. Our direct measurements of the Fermi surface further exclude a Lifshitz transition and associated divergence in the single particle density of states in the relevant doping range.

In Fig. 3a, b we quantify the spectral weight suppression in the PG state along the FS (see Supplementary Information B for details). This shows an extended ungapped region stretching out from the nodal point up to *θ* ≃ 13. 5° for *x* = 0.1 and *θ* ≃ 18° for *x* = 0.2, slightly larger than observed in overdoped Bi_2_Sr_2_CaCu_2_O_8+*δ*_^29^. Importantly, though, the PG sets on deep into the nodal lens pockets for both *x* = 0.1 (LD sample) and *x* = 0.2 (HD sample). Hence, no conventional closed Fermi surface emerges up to the highest doping of *x* = 0.2. Instead, we find disconnected Fermi arcs extending out from the node, as illustrated schematically in Fig. 3e.

Hsu et al. interpret the Hall carrier density crossover from *n*_H_ = 1 + *x* at high doping to *n*_H_ = *x* at low doping as a reconstruction of a conventional large FS (LFS) upon entering the PG phase. An ungapped LFS – illustrated in Fig. 3c – has a well-defined relation of Fermi wave vector *k*_F_ and carrier density per Ir *n* defined by the Luttinger theorem and shown as a black line in Fig. 3f. A simplified scenario for the PG phase is a small FS (SFS) consisting only of the lens-like nodal electron pockets while the antinodal hole pockets are gapped (Fig. 3d).

Our data question the applicability of these scenarios. Assuming a closed SFS appears arbitrary, given that the PG extends deep into the lens-like contours of the Fermi surface maps (Fig. 3a, b). Moreover, within a LFS scenario – commonly used in cuprates – our experimental *k*_F_ values for La concentrations 0.1 < *x* < 0.2 translate into carrier densities −0.09 < *n* < 0.07, nearly symmetric around zero doping.

A discrepancy of itinerant carrier densities and dopant concentration it not unusual. It can arise from co-doping from a slightly off-stoichiometric oxygen content or from a partial localization of doped electrons, as it is observed for instance in SrTiO_3_ 2D electron gases^30^. However, obtaining effective hole doping from substituting Sr by La is difficult to rationalize. Moreover, a LFS scenario places the evidently metallic state of the LD, *x* = 0.1 sample (Figs. 1c, 3a) at *n* ≃ 0 (half filling), which does not appear plausible. This suggests that the Luttinger theorem does not apply in the pseudogapped state of Sr_2−*x*_La_*x*_IrO_4_.

It further highlights that care must be taken when comparing doping values of iridates and cuprates. Applying a LFS scenario, all Sr_2−*x*_La_*x*_IrO_4_ samples studied in our work and by Hsu et al.^1^ are heavily underdoped in the sense that *n* is significantly smaller than optimal doping in cuprates (*p* ≃ 0.15). At the same time, our Sr_2−*x*_La_*x*_IrO_4_ samples with *x* ≃ 0.2 are overdoped in the sense that they have a large Hall carrier density *n*_H_ ≃ 1 + *x*^1^, which, in cuprates, is observed only above p* ≃ 0.19^7,8^.

### Temperature dependence

**Fig. 4:**
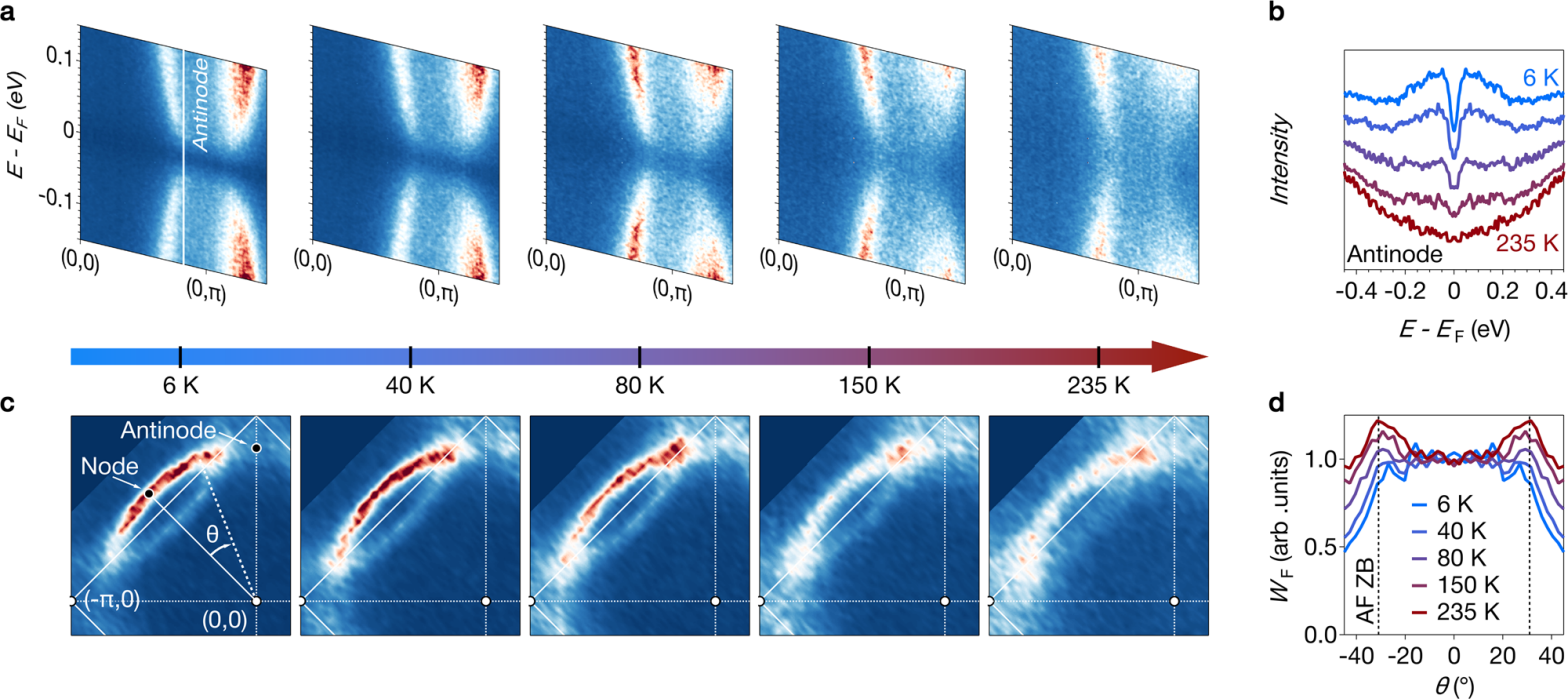
Temperature closure of the pseudogap for *x* = 0.2. **a** Temperature dependence of the symmetrized band dispersion in the antinodal direction. **b** EDCs at the antinode (offset for clarity). **c** Temperature evolution of the Fermi surface. **d** Temperature dependence of the spectral weight *W*_F_ along the Fermi surface. The angle *θ* is defined in (**c**). Data were averaged over ± *θ*. See Methods for the procedure used to extract *W*_F_. The dashed black lines shows the antiferromagnetic Brillouin zone boundary (AF ZB).

In Fig. 4a we show the temperature evolution of the antinodal spectral function at the highest doping *x* = 0.2 (*k*_F_ ≃ 0.67 Å). The clear gap, persisting all along the antinodal direction at *T* = 6 K, gradually becomes less pronounced as temperature increases and completely disappears by *T* = 235 K.

The EDCs at the antinodal *k*_F_, shown in Fig. 4b over a large energy range, suggest that the PG disappears primarily by a gradual loss of coherent spectral weight. Similar behavior was reported in cuprates below the critical hole doping p*^31^. Intriguingly, though, here it is observed at a doping above x* where the Hall carrier density is ≃ 1 + *x*^1^.

Measurements along the Brillouin zone diagonal (see Supplementary Information C) show that the nodal quasiparticle peak is more resilient and persists up to the highest temperature, albeit broadened. We finally note that low-temperature spectra taken immediately after the temperature-dependent measurements, as shown in Supplementary Information C, exhibit similar features to those observed before the temperature cycle. This rules out that the PG disappears in the data because of aging effects.

The vanishing PG further leads to a redistribution of spectral weight *W*_F_ along the Fermi surface (see Methods for the determination of *W*_F_). Figure 4d shows that the temperature dependence of *W*_F_ sets on abruptly at *θ* ≃ 18° where the ungapped Fermi arc ends. Increasing the angle further, *W*_F_ first shows a local maximum around *θ* ≃ 31° where the Fermi arc and its backfolded replica cross before it decreases towards the antinode. The suppression of weight towards the antinode is strongest at low temperature and gradually disappears as the highest temperature is approached, consistent with a vanishing PG. The temperature dependence of the PG further causes a slight shift in the angle of the precise position of the local spectral weight maximum.

We remark that the PG reported in Ref. ^23^ for surface K-doped Sr_2_IrO_4_ appears to be more fragile than the PG of bulk La-doped Sr_2−*x*_La_*x*_IrO_4_. In the K/Sr_2_IrO_4_ system the PG was observed to fully close at a K coverage of ~ 0.85 ML where *k*_F_ ≃ 0.67 Å. Our data show that at the same *k*_F_, the PG clearly remains open in bulk-doped samples. Moreover, the temperature boundary of T* ≃ 200 K found here for *x* = 0.2 is significantly higher than the maximal T* = 70 K–110 K at the K/Sr_2_IrO_4_ interface observed at a lower doping (coverage of 0.7 ML / *k*_F_ ≃ 0.655 Å). In addition, surface-doped Sr_2_IrO_4_ shows an apparent *d*-wave gap, suggesting potential high-temperature surface superconductivity^32,33^. Our data on bulk doped samples exclude a *d*-wave gap of similar magnitude and temperature dependence over the entire doping/*k*_F_ range studied in Refs. ^32,33^.

## Discussion

The temperature boundary of the PG in bulk electron-doped iridates observed here is strikingly similar to T* in cuprates^34,35^. This is intriguing considering the much lower on-site repulsion *U* and stronger spin-orbit coupling *λ*_SOC_ in iridates^19,36,37^ as well as the absence of superconductivity in the samples studied here^1,18^. On the other hand, iridates and cuprates show similar energy scales in the magnetic sector. Magnons in undoped Sr_2_IrO_4_ disperse up to ~205 meV at (*π*, 0)^19^, comparable to the ~ 320 meV in La_2_CuO_4_^38^. Moreover, spin fluctuations in both iridates^20,39^ and cuprates^40,41^ are known to persist up to high doping. These experimental findings point to an important role of short-range antiferromagnetic spin fluctuations in the pseudogap physics of Sr_2−*x*_La_*x*_IrO_4_. In turn, this provides further evidence for the importance of AF correlations for the PG in cuprates, which is also seen in numerical solutions of the Hubbard model^28,42–46^.

We further note a pronounced asymmetry between electron- and hole-doped iridates. A recent study of hole-doped Sr_1.93_K_0.07_IrO_4_ films found a conventional large FS, in sharp contrast to the PG state of Sr_2−*x*_La_*x*_IrO_4_^47^. This is reminiscent of differences observed in hole- and electron-doped cuprates, although it remains unclear whether the underlying origins are similar. Indeed, both the orbitals involved and the nature of the insulating ground state are different: Cu *e*_*g*_ and O 2*p* orbitals with a large charge-transfer gap for the cuprates, spin-orbitally entangled Ir *t*_2*g*_ orbitals with small putative Mott-Hubbard gap in the iridate. Consequently, in cuprates doped holes have a different orbital character than doped electrons^48^, while they have different internal degrees of freedom in iridates and thus a different motion in their local magnetic environment^49^.

In summary, our ARPES measurements of highly doped Sr_2−*x*_La_*x*_IrO_4_ samples show a smooth evolution of the electronic structure up to the highest doping of *x* ≃ 0.2. Most importantly, we find that the anisotropic PG of Sr_2−*x*_La_*x*_IrO_4_ persists up to *x* = 0.2 and thus beyond the crossover to a large Hall carrier density ≃ 1 + *x* reported by Hsu et al. at a critical doping x* ≃ 0.16. In cuprates, the crossover of the Hall density from ≃ *x* to ≃ 1 + *x* occurs at – or very close to – the critical doping p* where the PG ends^7,8^. Our work, taken together with the results of Hsu et al., shows that iridates differ in this crucial aspect of PG phenomenology. At high doping, a large Hall carrier density ≃ 1 + *x* coexists in iridates with an anisotropic pseudogap, suppressing the antinodal spectral weight. Understanding the nature of this unusual state will require further experimental and theoretical work.

## Methods

### Crystal growth

Single crystals with less than *x* = 0.11 lanthanum content (LD samples) were grown using a conventional flux cooling method, detailed elsewhere^15^. The lanthanum was measured by energy-dispersive X-ray spectroscopy and electron probe X-ray microanalysis. A flux evaporation method was used for crystals with high lanthanum concentrations (HD samples). For *x* = 0.2, SrCl_2_ (Alfa Aesar, anhydrous, 2N_5_) flux was combined with SrCO_3_ (Sigma Aldrich, 4N), IrO_2_ (Alfa Aesar, 3N) and La_2_O_3_ (Sigma Aldrich, 4N) in the ratio IrO_2_ + 8.5SrCl_2_ + 1.44SrCO_3_ + 0.36La_2_O_3_. The SrCO_3_ was dried at 600 °C and La_2_O_3_ at 1000 °C for 24 h. 0.83 g of IrO_2_ was used for the successful attempts. All materials were ground in an agate mortar and pestle for twenty minutes and loaded into a 30 mL platinum crucible with a loose platinum lid. The crucible was loaded into a standard box furnace at 700 °C, ramped to 1350 °C in 3 h and held for 30 h. The furnace was then cooled to 800 °C at 4 °C / min, the crucible was removed, and air quenched to room temperature.

The crucible held a mixture of phases, including large (<2 mm), square platelet samples (perhaps twenty per batch) and iridium metal. Little flux remained in the crucible, and the crystals were removed by soaking in warm water. There were often several morphologies, including triangular-platelet and square-platelet. Interestingly, the square-platelet crystals were consistently above *x* = 0.16 La content, while the triangular-platelet crystals were less than *x* = 0.11. The successful method was highly susceptible to synthesis conditions. Variations in starting mass, time baking, crucible volume, and temperature could all affect the outcome, preventing crystal growth of high La concentrations. The baking time needed to be long enough for the majority of the flux to evaporate, driving the growth of large crystals. The temperature window of successful growth was small, perhaps twenty degrees Celsius, vital to achieve high doping. Essentially, the intermediate oxidation state of the iridium required for high lanthanum doping is stabilized by temperature and chemical environment. If the temperature is too low, the iridium will not be sufficiently reduced, and if it is too high, the flux will evaporate too quickly, and the iridium will be reduced to metal. Successful reproduction of our method will require fine-tuning individual set-ups to optimize the temperature and starting mass/growth time.

### ARPES measurements

Angle-Resolved Photoemission Spectroscopy (ARPES) experiments were performed at the I05 beamline (Diamond Light Source), the BLOCH beamline (Max IV), and the SIS beamline (Swiss Light Source). Samples were cleaved in ultra-high vacuum conditions (*P* < 10^−10^ mbar) and at low temperatures, *T* ≃ 50 K for insulating samples (*x* = 0, 0.02), and *T* ≤ 20 K for conducting samples (*x* ≥ 0.1). The energy resolution ranged from 10 meV to 30 meV, depending on the specific measurement. The Fermi level was calibrated from reference measurements on a polycrystalline Au sample.

The data shown in Figs. 1, 2a, 3a, b, and 4 were acquired at the I05 beamline with 100 eV photon energy, linear horizontal (LH) polarization, a vertical analyzer slit, and an ARPES spot size of approximately 50 × 50 *μ*m^2^. The light red square and purple dot shown in Figs. 2b–d and 3f were obtained from these data.

The dark red square in Figs. 2b–d and 3f was extracted from data collected at the SIS beamline with 68 eV photon energy, circular right polarization, a horizontal analyzer slit, and a spot size of about 50 × 100 *μ*m^2^.

The measurements shown as the blue and green dots in Figs. 2b–d and 3f were performed at the BLOCH beamline, using 68 eV photon energy, LH polarization, a vertical analyzer slit, and a spot size of approximately 10 × 10 *μ*m^2^.

### Details on data analysis

The data from undoped and lightly doped samples (*x*≤0.1) shown in Fig. 1 are extracted from the dataset of Ref. ^15^. All the Fermi surface maps were obtained by integrating the measured intensity in the range *E*_F_ ± 5 meV.

Fermi momenta *k*_F_ and scattering rates at the Fermi energy *Γ*_F_, depicted in Fig. 2b–d, were obtained by fitting nodal Momentum Distribution Curves (MDCs) integrated over the range *E* = *E*_F_ ± 2.5 meV. The MDCs, extending over the entire Brillouin zone, were modeled using four Lorentzian peaks with a second-order polynomial background, convolved with a Gaussian profile of full width at half maximum *ΔE*/*v*_F_ where *ΔE* is the energy resolution and *v*_F_ the Fermi velocity. The Fermi velocity *v*_F_ in Fig. 2c was obtained from linear fits of the MDC peak dispersion between *E*_F_ − 30 meV and *E*_F_ − 10 meV. Where available, the values of *k*_F_, *Γ*_F_, and *v*_F_ extracted from both sides of the *Γ* points were averaged.

To determine the pseudogap area as a function of the angle *θ* in Fig. 3a, we first removed a second-order polynomial background defined far from *E*_F_ (typically from *E* − *E*_F_ = − 0.3 eV to *E* − *E*_F_ = −0.05 eV) to EDCs averaged over the range *k*_F_ ± 0.015 Å for every Fermi surface angle *θ*. The background-substracted symmetrized EDCs relative to *E*_F_ were fitted with a Gaussian profile, the area of which were averaged for ± *θ* and define the pseudogap spectral weight suppression.

The spectral weight at the Fermi level *W*_F_ shown in Fig. 4d was obtained by averaging the measured intensity in the range *E*_F_ ± 10 meV and *k*_F_ ± 0.015 Å at ± *θ* for $\theta \in \left[-4{5}^{\circ },4{5}^{\circ }\right]$. For each temperature *T*, this quantity was normalized by the average nodal spectral weight ${W}_{{\rm{F}}}(\theta \in \left[{0}^{\circ},\pm {5}^{\circ}\right],T)$.

## Supplementary information

Supplementary information

## Data Availability

The datasets analyzed during the current study are available at the Yareta repository of the University of Geneva^50^.
