# Supplementary material for: Fermi surface and pseudogap in highly doped Sr2IrO4
Source: NPJ Quantum Mater. 2025 Oct 3;10(1):100. doi: 10.1038/s41535-025-00817-9 (PMC12494496; doi:10.1038/s41535-025-00817-9)
Supplement: Supplementary file 1 — Supplementary information [file 41535_2025_817_MOESM1_ESM.pdf]

Supplementary Information for 'Fermi surface and pseudogap in highly doped Sr_2IrO_4'

Y. Alexanian,[1,*] A. de la Torre,[2,3] S. McKeown Walker,[1,4] M. Straub,[1] G. Gatti,[1] A. Hunter,[1] S. Mandloi,[1] E. Cappelli,[1] S. Riccò,[1] F. Y. Bruno,[5] M. Radovic,[6] N. C. Plumb,[6] M. Shi,[6,7] J. Osiecki,[8] C. Polley,[8] T. K. Kim,[9] P. Dudin,[9,10] M. Hoesch,[11] R. S. Perry,[12,13] A. Tamai,[1] and F. Baumberger[1,6]

[1]*Department of Quantum Matter Physics, University of Geneva, Geneva, Switzerland*
[2]*Department of Physics, Northeastern University, Boston, MA, USA*
[3]*Quantum Materials and Sensing Institute, Northeastern University, Burlington, MA USA*
[4]*Laboratory of Advanced Technology, University of Geneva, Geneva, Switzerland*
[5]*GFMC, Departamento de Física de Materiales, Universidad Complutense de Madrid, Madrid, Spain*
[6]*Swiss Light Source, Paul Scherrer Institut, Villigen, Switzerland*
[7]*Center for Correlated Matter and School of Physics, Zhejiang University, Hangzhou, China*
[8]*MAX IV Laboratory, Lund University, Lund, Sweden*
[9]*Diamond Light Source, Harwell Campus, Didcot, UK*
[10]*Synchrotron SOLEIL, Gif sur Yvette, France*
[11]*Deutsches Elektronen-Sychrotron DESY, Hamburg, Germany*
[12]*ISIS Pulsed Neutron and Muon Source, STFC Rutherford Appleton Laboratory, Harwell Campus, Didcot, UK*
[13]*London Centre for Nanotechnology and Department of Physics and Astronomy, University College London, London, UK*

A. SAMPLE CHARACTERIZATION AND DOPING DEPENDENCE

In Fig. S1, we present the evolution of the ARPES spectra of $Sr_{2-x}La_xIrO_4$ as a function of the ARPES spot position in one of our HD samples, which exhibits inhomogeneous chemical doping x. Spectra in the nodal direction measured at several points spaced by 30 µm (see Fig. S1a) are shown in Fig. S1b. Significant changes in the electronic structure near the Fermi energy are observed: the two branches around $(\pi/2, \pi/2)$, forming the lenses of the Fermi surface, are indistinguishable near the sample edge but become clearly visible towards its center. This evolution correlates with variations in the lanthanum concentration x along the line, as measured by energy-dispersive X-ray spectroscopy (EDX), and shown in Fig. S1c. Interestingly, we found that the Fermi momentum k_F (extracted from MDCs at $E = E_\mathrm{F}$) increases linearly with the chemical doping value x (Fig. S1d). Although the chemical doping x cannot be directly related to electron doping (see main text), its evolution in a single type of sample reflects changes in electron doping. Finally, note that this spatial variation of x is typical for our HD samples, whereas LD samples exhibit a more homogeneous doping distribution.

B. ANGULAR DEPENDENCE OF THE PSEUDOGAP

In Fig. S2 we provide additional details on the determination of the angular dependence of the pseudogap shown in Figs. 3a, 3b of the main text. First, we fitted a second-order polynomial background $B(E-E_\mathrm{F})$ in the energy interval $-0.3\,\mathrm{eV} \leq E-E_\mathrm{F} \leq -0.05\,\mathrm{eV}$ of the EDCs measured at k_F for various angles θ (each EDC corresponds to a color dot in Figs. 3a, 3b). We then removed the background of the EDCs and fitted the symmetrized remaining signal in the energy range $-0.05\,\mathrm{eV} \leq E-E_\mathrm{F} \leq 0.05\,\mathrm{eV}$ with a Gaussian function defined as:

$$G(E-E_\mathrm{F}) = \frac{A}{2\gamma\sqrt{\pi/\ln(16)}}\exp\left(-\left(\frac{E-E_\mathrm{F}}{2\gamma}\right)^2\ln(16)\right). \tag{S1}$$

Examples of the EDCs and their total fits $B(E-E_\mathrm{F})+G(E-E_\mathrm{F})$ are shown in Fig. S2a for $x = 0.1$ and Fig. S2b for $x = 0.2$. The spectral weight suppression in the PG, shown in Figs. 3a, 3b of the main text, is defined as the area A. Figs. S2c-e show the area A together with the gap width γ, and the gap amplitude A/γ. Interestingly, our fits also indicate that it is the gap amplitude, rather than the gap width, that decreases as the node is approached. Lastly, note the presence of a local maximum (and corresponding local minimum) in the gap area (gap width) around $\pm 30°$, where the primitive and backfolded bands cross.

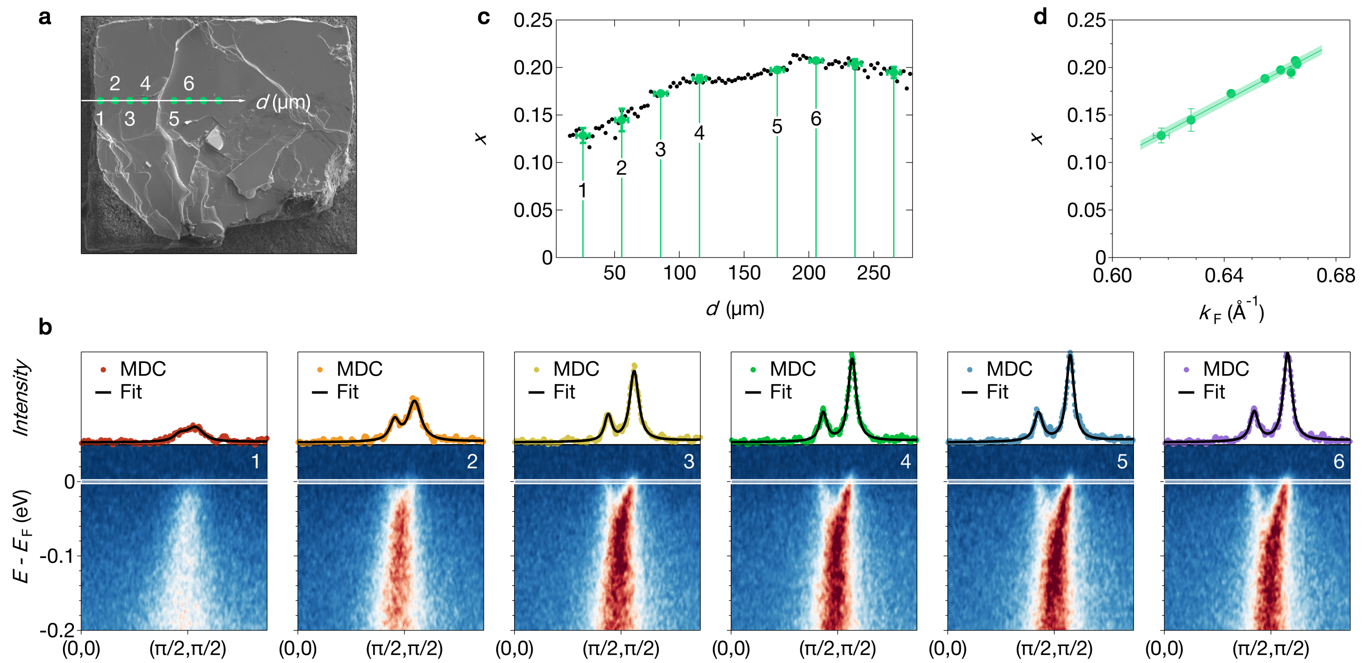

FIG. S1. **Spatial dependence of the ARPES spectra measured in an HD sample.** **a** Scanning electron microscope image of a cleaved sample. The green dots represent the position and size of the ARPES spots at different measurement points. The spot size was approximately 10 μm, and the points were spaced by 30 μm along a line (in white). **b** ARPES cuts and MDCs at the Fermi energy (integrated over the range $E = E_F \pm 2.5\,\mathrm{meV}$) along the nodal direction measured at different positions on the sample, as shown in panel (**a**). MDCs are fitted with two Lorentzian peaks and a second-order polynomial background convolved with a Gaussian profile of full width at half maximum determined by the energy resolution. **c** EDX characterization of the doping value x along the line of the ARPES measurements indicated in panel (**a**). Green lines indicate positions of the ARPES measurements. Green markers give doping values averaged over the beam spot diameter of 10 μm with standard deviations. **d** Fermi momentum k_F with standard deviations (often smaller than the marker size) extracted from the MDCs as a function of the chemical doping value x obtained from EDX measurements. The green line is a linear fit to the data.

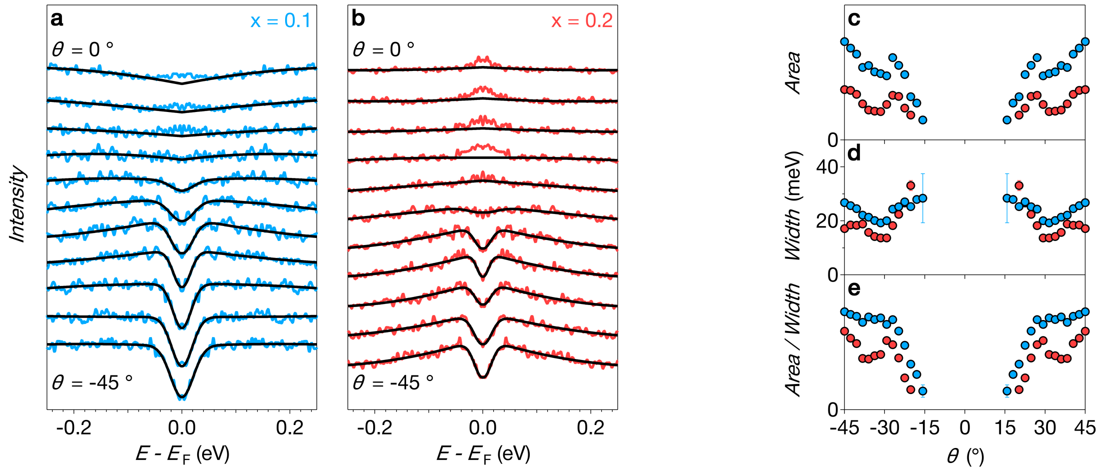

FIG. S2. **Angular dependence of the $Sr_{2-x}La_xIrO_4$ pseudogap for $x = 0.1$ and $x = 0.2$.** **a,b** Fits of the symmetrized EDCs (with respect to E_F) measured at k_F with an angle θ compared to the nodal direction. The fitting procedure is described in the supplementary text. **c** Pseudogap area A, **d** pseudogap width γ, and **e** pseudogap amplitude A/γ as a function of the angle θ compared to the nodal direction. Values obtained for angles $+\theta$ and $-\theta$ were averaged. Error bars represents the standard deviation of the fitted parameters, and are smaller than the marker size except for few data points.

C. COMPLEMENT ON THE TEMPERATURE DEPENDENCE

In Fig. S3 we present temperature-dependent measurements of the electronic structure of $Sr_{1.8}La_{0.2}IrO_4$ along the nodal direction. Symmetrized cuts from $T = 6$ K to $T = 235$ K (see Fig. S3a) reveal the absence of any gap within this temperature range, in stark contrast to the temperature-dependent pseudogap observed in the antinodal direction (see Fig. 4a of main text). Moreover, the nodal EDCs at different temperature, shown in Fig. S3b, demonstrate that the sharp quasiparticle peak visible at $T = 6$ K broadens with increasing temperature but remains present up to the highest temperature measured. Notably, we observe no significant change when the pseudogap vanishes (between $T = 150$ K and $T = 235$ K).

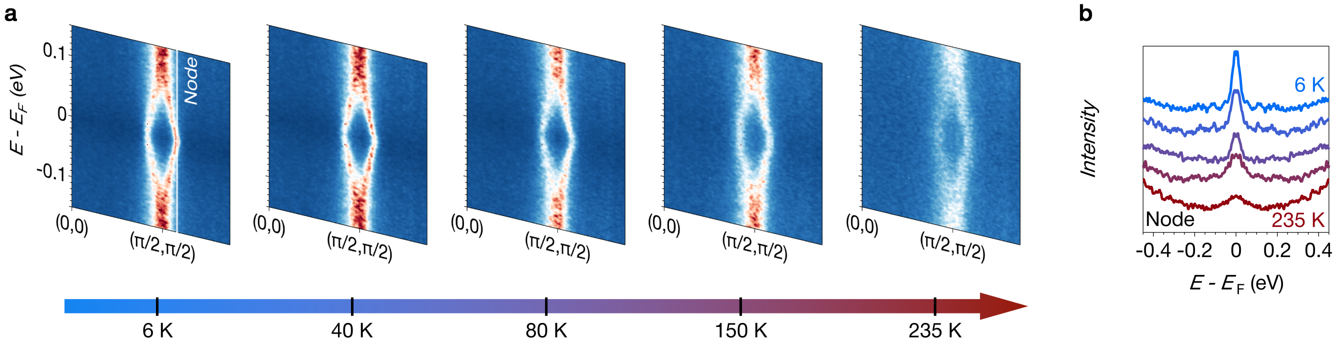

FIG. S3. **Temperature dependence of nodal ARPES spectra in $Sr_{1.8}La_{0.2}IrO_4$. a** Temperature dependence of the symmetrized band dispersion in the nodal direction. The node position is indicated by the white line. **b** EDCs at the node, shifted by a different constant intensity for each temperature.

Low-temperature measurements ($T = 20$ K) taken after the complete temperature cycle from $T = 6$ K to $T = 235$ K are shown in Fig. S4. Symmetrized cuts along both the nodal and antinodal directions (Figs. S4a, S4b) as well as the Fermi surface (Fig. S4c) are similar to those obtained at low temperature before the cycle (see Figs. 4 and S3). Most importantly, a pseudogap remains evident at the antinode, while no gap is observed at the node, ruling out any aging effect as an explanation for the pseudogap closure in our data.

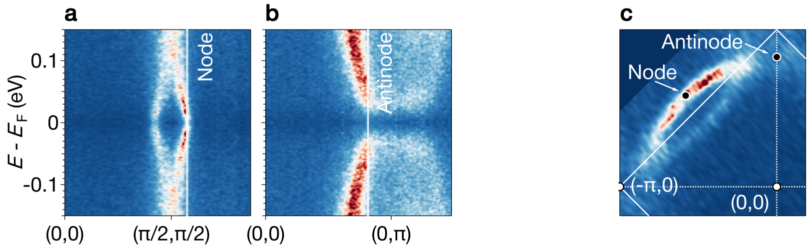

FIG. S4. **Low-temperature measurements of $Sr_{1.8}La_{0.2}IrO_4$ after a complete temperature cycle. a,b** Symmetrized cuts along the nodal and antinodal directions, respectively. **c** Fermi surface.

* yann.alexanian@unige.ch
